# Disease burden and healthcare utilization in pediatric low-grade glioma: A United States retrospective study of linked claims and electronic health records

**DOI:** 10.1093/nop/npae037

**Published:** 2024-04-27

**Authors:** Susan Zelt, Tabitha Cooney, Sandie Yu, Shailaja Daral, Blake Krebs, Riddhi Markan, Peter Manley, Mark Kieran, Sandya Govinda Raju

**Affiliations:** Day One Biopharmaceuticals, Inc., Brisbane, California, USA (S.Z., T.C., S.Y., P.M., M.K., S.G.R.); Day One Biopharmaceuticals, Inc., Brisbane, California, USA (S.Z., T.C., S.Y., P.M., M.K., S.G.R.); Day One Biopharmaceuticals, Inc., Brisbane, California, USA (S.Z., T.C., S.Y., P.M., M.K., S.G.R.); Optum Lifesciences, Inc., Eden Prairie, Minnesota, USA (S.D., B.K., R.M.); Optum Lifesciences, Inc., Eden Prairie, Minnesota, USA (S.D., B.K., R.M.); Optum Lifesciences, Inc., Eden Prairie, Minnesota, USA (S.D., B.K., R.M.); Day One Biopharmaceuticals, Inc., Brisbane, California, USA (S.Z., T.C., S.Y., P.M., M.K., S.G.R.); Day One Biopharmaceuticals, Inc., Brisbane, California, USA (S.Z., T.C., S.Y., P.M., M.K., S.G.R.); Day One Biopharmaceuticals, Inc., Brisbane, California, USA (S.Z., T.C., S.Y., P.M., M.K., S.G.R.)

**Keywords:** burden of illness, CNS tumors | healthcare resource utilization (HRU), pediatric low-grade glioma, pediatric oncology

## Abstract

**Background:**

Despite high long-term survival rates, pediatric low-grade gliomas (pLGGs) are linked with significant tumor- and treatment-associated morbidities that may persist throughout life. The aims of this descriptive cross-sectional pilot study were to characterize health conditions among a cohort of patients with pLGG and explore the feasibility of quantifying disease burden and healthcare resource utilization (HRU).

**Methods:**

Optum^®^ Market Clarity Data were used to identify patients aged ≤18 years with an ICD-10 code for brain neoplasm, ≥1 physician notes, and with evidence of pLGG recorded between January 1, 2017 and June 30, 2018. Outcomes including health characteristics, HRU, medications, and procedures were assessed at 6-month intervals over 36 months.

**Results:**

One hundred and fifty-four patients were identified with pLGG and over half experienced headache/migraine, respiratory infection, pain, or behavioral issues during the 36-month study period. The most common comorbidities were ocular/visual (including blindness), mental health disorders, seizures, and behavioral/cognition disorders. Most symptoms and comorbidities persisted or increased during the study period, indicating long-term health deficits. HRU, including speciality care visits, filled prescriptions, and administered medications, was common; 74% of patients had prescriptions for anti-infectives, 56% antiemetics, and 52% required pain or fever relief. Sixty-five percent of patients underwent treatment to control their pLGG, the most common being brain surgery. Little decline was observed in medication use during the study period.

**Conclusions:**

Patients with pLGG have complex healthcare needs requiring high HRU, often over a long time. Patients need to be optimally managed to minimize disease- and treatment-related burden and HRU.

Pediatric low-grade gliomas (pLGGs) are the most common brain tumors in children, accounting for approximately 30% of all childhood central nervous system (CNS) tumors in the United States (US).^1,2^ According to the 2021 WHO classification of tumors of the CNS,^3^ pLGGs are low grade (typically grade 1 or 2), heterogeneous malignancies that may be classified into 3 of 6 tumor families under the “glioma, glioneuronal and neuronal tumor” umbrella: pediatric type diffuse LGGs, circumscribed astrocytic gliomas, and glioneuronal and neuronal tumors.^3,4^ Long-term survival for patients with pLGG is favorable, with a 10-year survival rate of 95% for pilocytic astrocytomas and 86% for other LGG subtypes reported in the US.^5^

Despite generally favorable survival outcomes, the morbidity associated with pLGG is significant and chronic, with patients experiencing ongoing disease burden and significant late effects that may persist throughout life.^1,6–9^ Long-term morbidities are either tumor- or treatment-related and include neurocognitive deficits, cerebrovascular disease, secondary neoplasms, endocrine abnormalities, and psychological and social impacts.^6,10–13^ Tumor-related morbidities often include seizures, hemiparesis, focal neurological findings, behavioral changes, visual disturbances, cognitive dysfunction, and endocrine dysfunction.^6,9,14^ Treatment-related morbidities vary according to the treatment employed. Treatment options for pLGG have evolved in recent decades, and typically include surgery, chemotherapy, molecularly targeted therapies, radiotherapy, and/or a “watch and wait” approach.^1,15^ Lesions within the cerebral and cerebellar hemispheres are more amenable to gross total surgical resection,^16^ and surgery can lead to an 8-year overall survival rate of >95%.^17^ However, surgery is known to increase morbidity risk, often leading to cognitive, adaptive, and motor function deficits.^6,18,19^ In many instances, especially when complete resection is not possible, tumor recurrence remains an ongoing complication, necessitating further treatment.^20,21^ Chemotherapy, often a combination of vincristine and carboplatin, is the preferred first-line treatment for surgically complex cases, and the second-line treatment option postsurgery.^7^ However, chemotherapy has a high relapse rate and multiple regimens are frequently needed.^20^ Toxicity and tolerability issues with chemotherapy in infants and young children are a serious concern and include infection, myelotoxicity, ototoxicity, gastrointestinal symptoms, neuropathy, allergic reaction, hair loss, and psychological impacts.^22–25^ Therapies targeting key molecular alterations are also gaining prominence in pLGG treatment. They present with unique toxicity profiles, including cardiac dysfunction, ocular effects, dry skin, diarrhea, rash, and creatine phosphokinase elevation.^7,26,27^ Radiotherapy is largely reserved for older patients and when other treatment options have been exhausted.^7^ The most common side effects of radiation therapy are cognitive dysfunction, endocrinopathy, vasculopathy, and secondary malignancies with all of these issues known to persist into adulthood.^7,12,28^

The limited studies on the long-term burden associated with pLGG support the notion that patients with pLGG can experience chronic health deficits that may impact their quality of life (QOL) and socioeconomic outcomes.^11,13,29^ To our knowledge, a study of health resource utilization (HRU) in pLGG has not been reported. A better understanding of the influence of short- and long-term tumor- and treatment-related morbidities should facilitate treatment decisions and be of value in establishing much-needed treatment guidelines. Here, using medical claims and electronic health records (EHRs), we aim to characterize the health conditions among a cohort of US-based patients with pLGG by piloting an approach to understand their HRU over a 3-year period. Additionally, this study aims to explore the feasibility of quantifying burden in terms of HRU.

## Materials and Methods

### Study Design

We conducted a pilot cross-sectional, retrospective analysis using the Optum^®^ Market Clarity Data (de-identified) to assess administrative medical and pharmacy claims and EHRs of patients with pLGG. Patients aged ≤18 years with an International Classification of Disease, 10th Revision (ICD-10) diagnosis code for brain neoplasm (Supplementary Table 1) on claims or EHRs and ≥1 physician notes during the inclusion period (January 01, 2017–June 30, 2018) were evaluated (Supplementary Figure 1). For inclusion, patients had to have evidence of glioma histology and LGG tumor in structured data or evidence of glioma histology and LGG tumor in unstructured data (physician notes). Natural language processing (NLP) was used to identify pLGG-relevant data from unstructured data and to subsequently normalize, validate, and integrate them into the study cohort (Supplementary Table 2). Patients must have had continuous health plan enrollment with both medical and pharmacy coverage for ≥3 months at baseline prior to the index date (first claim or EHR with an ICD-10 code for brain neoplasm) during the inclusion period and ≥36 months after the index date (follow-up period), or continuous clinical activity (as evident from healthcare visits) for ≥3 months prior to the index date (baseline period) and the ≥36-month follow-up period. Patients with an ICD-10 diagnosis code for nonbrain neoplasms were excluded.

### Ethics Statement

Ethical approval is not applicable. This study was conducted entirely using de-identified EHR and claims data obtained from an existing database (Optum^®^ Market Clarity) and did not involve the collection, use, or transmittal of individually identifiable data. All database records are statistically de-identified and certified to be fully compliant with US patient confidentiality requirements set forth in the Health Insurance Portability and Accountability Act of 1996 (HIPAA). As the database used in the study is compliant with the HIPPA, this study was exempted from Institutional Review Board approval.

### Data Source

The Optum^®^ Market Clarity Data includes data of ~73 million patients across the US and Puerto Rico from January 2007 to June 2021. The database covers commercial and Medicare Advantage beneficiaries in claims, commercial, Medicare, and Medicaid beneficiaries in EHRs, including patients enrolled with either United Health Care or other networks that have data and research agreements with Optum^®^. The Market Clarity Data links EHR data with historical, linked administrative claims, pharmacy claims, physician claims, and facility claims (with clinical information) data and is inclusive of medications prescribed and administered. Clinically rich and specific data elements are sourced from the EHRs, including laboratory results, vital signs and measurements, diagnoses, procedures, and information derived from unstructured clinical notes using NLP.

### Outcomes

The following baseline demographics were collected: age, biological sex, race, and insurance type. Health characteristics and HRU variables were reported at baseline (preindex period) and throughout the study period at 6-month intervals (postindex period). These included: comorbid or coexisting conditions (identified by ICD-10 codes); clinical features/signs, diseases, and symptoms (SDS) terms (verbatims of patients or examination findings of physicians that are mentioned in physician notes, categorized by clinical experts); healthcare professional (HCP) provider speciality and their place of service; office or outpatient visits; emergency room (ER) visits; inpatient stays (including length of stay); procedures of interest (determined by Healthcare Common Procedure Coding System [HCPCS] and Current Procedural Terminology [CPT] codes); and medications (determined by filled prescriptions with HCPCS and National Drug Code [NDC] codes). Medications of interest were categorized on 2 levels: pharmacological class and pLGG-relevant medication. Medication relating to pLGG could include medications to treat pLGG or required to treat the symptoms of the disease or to relieve the side effects of pLGG treatments. Procedures of interest were further categorized by line of therapy.

### Statistical Analysis

Descriptive summary statistics (mean, median, and standard deviation) were used to describe the study population. Frequencies and percentages were used to describe reasons for HRU, comorbidities or coexisting conditions, healthcare providers consulted, place of healthcare services, filled prescriptions, administered medications, pLGG therapy, and lines of therapy. Descriptive summary statistics (mean, 5th, 25th, 75th, and 95th percentile) were used to describe the length of inpatient and ER stays. The number of missing responses for any given variable was reported for each variable assessed. Percentages were calculated using the total number of nonmissing responses as the denominator. Missing responses and unknown data were not included in any denominator count. Certain factors were unable to be quantified due to the nature of the dataset, including tumor histology, genomics, and anatomical location. These are further discussed in the limitations section.

## Results

### Patient Demographics

Of 2841 patients in the database who complied with the study eligibility criteria, 154 patients were identified as having pLGG and continuous insurance coverage during the inclusion period and were selected as the study cohort (Supplementary Figure 2). The median age of patients was 11 years, 49% were female, and 75% were non-Hispanic white (Table 1). Eighty-six patients (56%) received commercial benefits and 68 patients (44%) received Medicaid benefits.

**Table 1.** Baseline Characteristics

Characteristic	*N* = 154
Age at index date, years	
Median (range)	11 (2–18)
Age group classification at index date, years, *n* (%)	
<1	0
1–3	8 (5)
4–5	15 (10)
6–9	41 (27)
10–12	29 (19)
13–15	34 (22)
16–18	27 (18)
Sex, *n* (%)	
Male	78 (51)
Female	76 (49)
Race, *n* (%)	
White	116 (75)
Hispanic	20 (13)
Black	7 (5)
Asian	1 (<1)
Other/unknown	10 (6)
Insurance type, *n* (%)	
Commercial	86 (56)
Medicaid	68 (44)

### Health Characteristics

**Figure 1.**
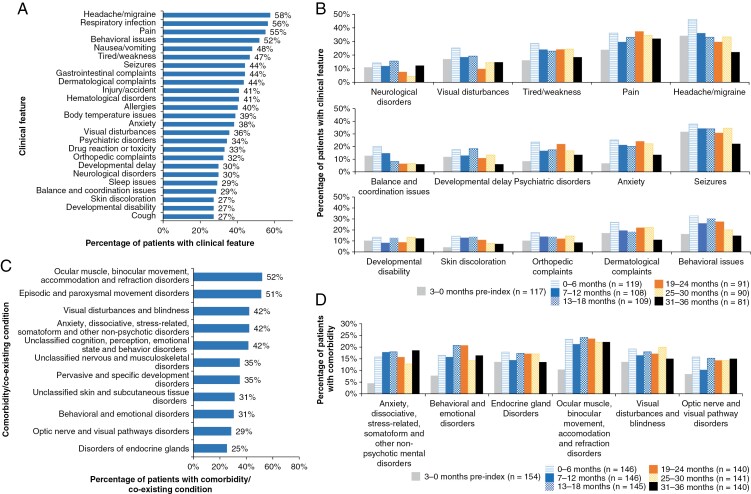
The most common clinical features in (A) the overall study period (*n* = 154) and (B) 6-month intervals*; select coexisting comorbidities in (C) the overall study period (*n* = 154) and (D) 6-month intervals*. Abbreviations: EHR: electronic health record; pLGG: pediatric low-grade glioma. *The *n* number for each time period indicates the number of patients in the study cohort that had ≥1 EHR of symptoms and signs of pLGG for that time period.

The most common signs and symptoms, excluding tumors and/or brain masses, in the overall study were headache/migraine (58%), respiratory infection (56%), and pain (55%) (Figure 1A). At baseline, the most common symptoms were headache/migraine (34%), seizures (32%), and respiratory infection (30%) (Figure 1B). Most reported clinical features tended to occur in higher proportions of patients during the follow-up period compared with baseline; for example, at 0–6 months postindex, headache/migraine, seizures, and pain occurred in 46%, 38%, and 36% of patients, respectively. These 3 clinical features remained common in the latest period (31–36 months postindex), affecting 22%, 22%, and 32% of patients, respectively. More than half of patients demonstrated behavioral issues (52% during the overall study period) which were more prominent in the follow-up period compared with baseline (16% in the preindex period versus a peak at 33% within the first 6 months postindex). Over a third experienced anxiety or a psychiatric disorder (38% and 34%, respectively) at some point in the study period. Nausea/vomiting (48%), fatigue (47%), gastrointestinal issues (44%), dermatological complaints (44%), hematological disorders (41%), and allergies (40%) were all also relatively common clinical features experienced by >40% during the study period.

The most common coexisting conditions/comorbidities in the overall study period, occurring in approximately half of all patients were ocular muscle, binocular movement, accommodation and refraction disorders (52%), and episodic and paroxysmal movement disorders (51%) (Figure 1C). The proportion of patients with comorbidities tended to be higher during the follow-up period compared with the baseline period (Figure 1D). In general, comorbidities remained consistent throughout the follow-up period with no obvious peaks and no clear pattern of decline or increase over time. (Figure 1D).

### Healthcare Providers and Service Utilization

**Figure 2.**
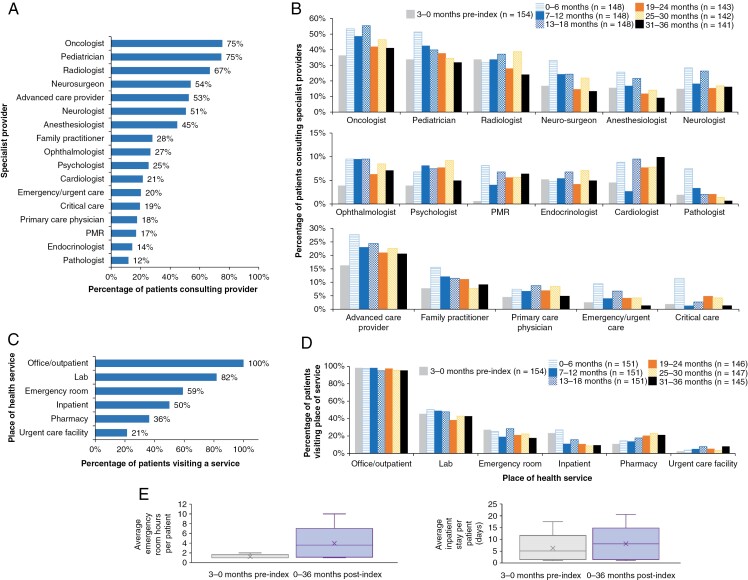
Healthcare specialist providers consulted in (A) the overall study period (*n* = 154) and (B) over time in 6-month intervals*. Locations of healthcare services in (C) the overall study period (*n* = 154) and (D) in 6-month intervals*;and (E) mean inpatient^†^ and emergency room^†^ stay durations. Abbreviation: PMR: physical medicine and rehabilitation. *The *n* number for each time period indicates the number of patients in the study cohort that had ≥1 claim or EHR for that time period. ^†^Inpatient stays: 3-month preindex (*n* = 36); 36-month postindex (*n* = 74); emergency rooms stay: 3-month preindex (*n* = 42); 36-month postindex (*n* = 84).

The most commonly reported pLGG-specific provider specialities in the overall study period were oncologists (75%), pediatricians (75%), radiologists (67%), neurosurgeons (54%), and advanced care providers (53%) (Figure 2A). The most common period for specialist consulting was the first 6 months, where the proportion of patients consulting pediatricians, neurosurgery specialists, pathologists, and critical care specialists (among others), was the highest (Figure 2B). Several of the reported speciality providers demonstrated substantial variability over the study period, including ophthalmologists and physical medicine and rehabilitation (PMR) specialists, who were reported for 4% and 1% of patients at baseline versus 9% and 8% of patients at 0–6 months follow-up. Additionally, the proportion of patients seeing a critical care specialist during the first 6 months was substantially higher than any other observation interval (11% at 0–6 months versus 1–5% in other intervals) (Figure 2B).

The most common locations of HRU during the study period were office/outpatient (100%) and laboratory (82%) settings (Figure 2C). Most patients (95–98% across the study period) visited an office or outpatient facility, 38–50% a diagnostic laboratory, 18–28% an ER, and 9–27% an inpatient facility in each of the 6-month intervals (Figure 2D). Urgent care facility use was generally low (3–8% across the study period) but was at the highest value at 31–36 months. The proportion of patients visiting pharmacies tended to increase over the follow-up period, while inpatient stays, after an initial increase between baseline and 0–6 months postindex, tended to decrease over the follow-up period. However, the mean inpatient stay duration increased by 59% from 5.1 days during the baseline period to 8.1 days during the follow-up period (Figure 2E). Additionally, mean ER stays increased from 1.3 h baseline to 3.6 h during the follow-up period.

### Medications and Procedures

**Figure 3.**
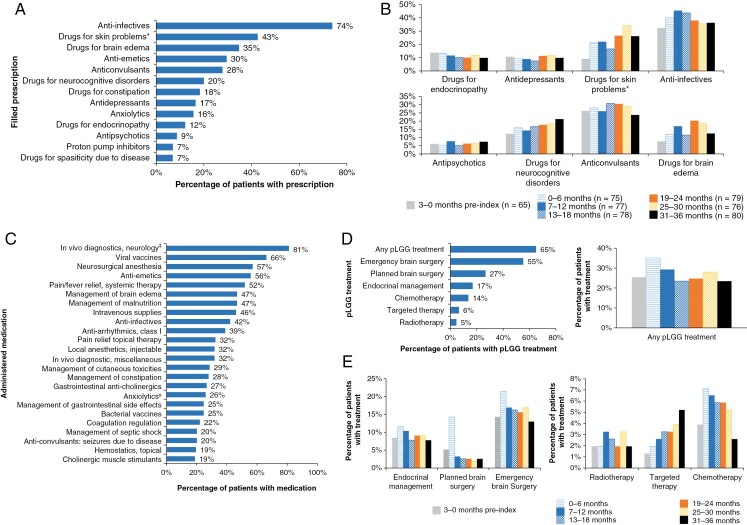
Filled prescriptions in (A) the overall study period (*n* = 154) and (B) over time in 6-month intervals (the *n* number for each time period indicates the number of patients in the study cohort that had ≥1 EHR of symptoms and signs of pLGG for that time period); (C) administered medications in the overall study period; (D) pLGG therapy subtype use in the overall study period and any pLGG therapy use in 6-month intervals*; and (E) pLGG therapies by subtype over time (D/E: *n* = 154 for all time periods). pLGG, pediatric low-grade glioma. *Includes management of cutaneous toxicities due to targeted therapy. ^‡^Gabobutrol or gadodiamide sodium administration for imaging. ^#^Neuropsychological comorbidity due to disease.

The most common filled prescriptions in the overall study period included those for anti-infectives (74%), acne treatment including management of cutaneous toxicities due to targeted therapy (43%), drugs for management of brain edema (35%), and antiemetics (30%) (Figure 3A). Anti-infectives were used consistently throughout the study period, with more than 30% of patients requiring them during each observation interval. Other drugs commonly prescribed in this analysis (≥20% of patients) included anti-convulsants (28%) and drugs for neurocognitive disorders (20%). The proportion of patients requiring drugs for skin problems, brain edema, and neurocognitive disorders tended to increase over time during the follow-up period, while the proportion of patients requiring anti-depressants, anti-convulsants and drugs for endocrinopathy remained at similar levels throughout the study period (Figure 3B).

The most commonly administered medications in the overall study period were contrasted imaging agents as part of neurology in vivo diagnostics (81%, comprising gadobutrol or gadodiamide sodium administration), viral vaccines (66%), neurosurgical anesthesia (57%), antiemetics (56%), and pain/fever relief (52%) (Figure 3C). The administration of viral vaccines increased from 9% in the baseline period to 24–51% in the follow-up period. The use of neurosurgical anesthesia, antiemetics, and pain/fever relief medications increased substantially between the baseline and first 6-month observation interval (23% to 47%, 21–42%, and 19–35%, respectively).

### pLGG Treatment

In the overall study period, 65% of patients received pLGG-specific treatment, the most common being emergency brain surgery (55%) and planned brain surgery (27%) (Figure 3D). Most patients received planned brain surgery in the first 6-month observation interval, while emergency brain surgery was more evenly distributed throughout the follow-up period. Therapeutic management of endocrinal complications was required for 17% of patients, with its use remaining stable throughout the study period. Chemotherapy was given to 14% of patients during the study, with the highest proportion of patients receiving it in the first 6-month observation interval (7%) and its use declining over the follow-up period. Though the overall proportion of patients receiving off-label targeted therapies was low (6%), their use escalated during the follow-up period, increasing from 1% at baseline to 5% at 31–36 months. Use of radiotherapy was low in this cohort (5%) and remained at 3% or lower during each observation interval.

Of 100 evaluable patients, 43% had received 1 line of pLGG-specific therapy, 37%, 2 lines of therapy, and 20%, 3 or more lines of therapy, during the study period.

## Discussion

In this retrospective claims-based pilot study of 154 US patients with pLGG, not only were high levels of HRU observed during the 3-year study period, but healthcare needs persisted over time with little improvement. The HRU identified in this analysis highlights the chronic burden on patients, families, and the healthcare system. This is our first attempt to characterize the burden of pLGG in the context of HRU and provide a snapshot of the challenges faced by patients with pLGG. While they live longer than children with more aggressive tumors, patients with pLGG suffer from a high burden of HRU given the chronic nature of their underlying disease and the impact of multiple treatments.

pLGG is heterogeneous in terms of tumor location, and a range of comorbidities will often occur at different time points throughout the disease process. Our observations corroborate with previous reports on common comorbidities such as visual disturbances, seizures, fatigue, pain, and headaches.^30–32^ One example is seizures, experienced by 44% of patients in this analysis throughout the study period—this observation over a finite observation interval appears consistent with the seizure rates reported for all patients with LGGs (60–85%) at some point in the course of their disease compared with a rate of 1–2% experienced in the general population.^31^ Our findings are also consistent with reports in general pediatric oncology populations where high levels of HRU, due to long-term disease- and treatment-related morbidities, led to negative long-term impacts on neurodevelopment and mental health.^33–36^ A recent systematic review and meta-analysis of 52 pediatric oncology-controlled trials by Lee et al. found that children and adolescents with cancer had an increased risk of depression, anxiety, and psychotic disorders postcancer remission versus siblings and healthy matched controls.^33^ Consistent with these findings, our analysis found HRU in more than half of patients for behavioral health-related issues and over a third for anxiety or psychiatric disorders. A pLGG-specific retrospective cohort analysis of 45 survivors (from birth to 16 years at diagnosis) where 66% self-reported at least 1 neurological-related issue and 57% self-reported cognitive deficits also aligns with our findings; this analysis found that the pLGG cohort self-reported poorer QOL, worse academic performance, and reduced cognitive functioning versus available statistics from similar populations, warranting additional tailored surveillance and preventative or curative interventions from healthcare providers.^32^ Other commonly reported concerns in this pLGG cohort included headaches (47% in young adults), fatigue (29–30% in young children, adolescents, and young adults), and pain (29% in young children), consistent with our findings.^32^ Although our analysis lacks a matched comparator, a previous study has shown that when compared with their healthy counterparts, survivors of pediatric brain tumors often experience impaired fine motor skills and reduced cognitive functioning.^37^

Patients with pLGG require a specialized multidisciplinary healthcare team that may change as their disease, and management of it evolves—as evidenced by the variability in HCP involvement observed in our analysis. Many patients consulted with various specialists throughout the study period, but the first 6-month observation interval typically involved the most specialities, which is expected during the acute management phase of pLGG. Neurosurgeons are often the first specialists consulted during the patients’ journey as discussions regarding surgical options are often the first step following diagnosis of pLGG. It should be noted that pLGG may also be handled differently by various institutions; for example, resected-only tumors may primarily be managed by neurosurgery versus referral to other institutes for more comprehensive neuro-oncology care in more complex cases. Ideally, any patient who receives an oncology-related diagnosis should receive supportive care throughout their disease and posttreatment, including psychosocial support, management of more immediate as well as long-term and late effects, and potentially specific types of rehabilitation.^38^ Results from this study suggest similarities with pLGG as ongoing supportive care was needed throughout the study period involving PMR specialists, psychologists, ophthalmologists, and advanced care providers. As this analysis was claims-based and was not designed to collect information on the ease of access to specialists and supportive care by patients with pLGG, future studies could assess disease/treatment-related burden and HRU more comprehensively.

In this study, compared with the baseline period, the average per-patient time spent in the ER more than doubled and inpatient stays increased by 59% over the follow-up period. This is not an unexpected trend in the inpatient setting, as treatment, often surgery, is initiated first. However, the increase in time spent in the ER, including the need for emergency neurosurgery, suggests a lack of well-controlled disease or may be attributable to other variables (ie a shunt malfunction). Time spent in ER visits and inpatient stays places a substantial burden on families and adds to time spent away from work, school, and normal activities, potentially adding to financial burden and overall stress.

The majority of medications and procedures reported either increased or remained the same as baseline throughout the follow-up period. The consistent need for medication observed in our study suggests an ever-present burden on patients and their families for healthcare visits and adherence to treatment. Anti-infectives were the most filled prescription overall and also the most common prescription in each 6-month observational interval, suggesting a persistent risk of infection and long-lasting immunosuppression induced by treatment. To date, there is no consensus on the prophylactic use of antimicrobial agents in children with pLGG. Other drugs commonly prescribed in this analysis included those for skin problems, edema, neurocognitive disorders, nausea/vomiting, and seizures, reflecting the range of complications affecting children with pLGG, including toxicities associated with the use of chemotherapy, radiotherapy, and targeted therapy. Our data aligns with previous studies reporting on the increased medication burden among survivors of pediatric cancer which highlighted the increased rates of prescriptions, particularly within the first 3 years posttherapy.^39–41^

Almost two-thirds of this cohort received some type of pLGG-specific treatment to control their disease. Planned and emergency surgery occurred most commonly within the first 6-month observation interval. However, while planned surgery declined over time, emergency surgery remained stable throughout the follow-up period. Further data would be needed to fully understand the role of emergency surgery in pLGG observed in our analysis (ie shunt malfunctions versus rapid disease progression). After surgery, treatment for endocrine abnormalities was the most commonly reported pLGG treatment, which is consistent with published reports.^8,42–44^ A pLGG-specific retrospective analysis (*n* = 814) reported 1 or more endocrinopathies in 11% of patients, with an increased risk of developing multiple endocrinopathies associated with chemotherapy and radiotherapy.^42^ A retrospective study reported similar findings in survivors of CNS tumors (*n* = 1877) where a significantly higher risk of developing an endocrine condition 5 years after diagnosis was observed when compared with siblings.^8^

Multiple treatment modalities, including the advent of new therapies, introduce additional challenges in patient care such as novel treatment-related effects, late effects, and accumulation of toxicity.^15^ The use of chemotherapy, radiotherapy, and targeted therapy observed in our study was relatively low. This suggests that some patients may have been treated with surgery only and did not require additional pLGG treatments or that a number of patients were placed on a “watch and wait” approach, which is not easily captured by a claims-based analysis. The low use of radiotherapy is expected in this pediatric setting given its impact on the developing CNS and associated risks.^12^ Use of targeted therapies increased throughout the study period, peaking at 5% by 31–36 months. Targeted therapies are newer to the pLGG treatment landscape (off-label during the timeframe of this study) and adoption was possibly slower. Targeted therapies are typically reserved for refractory cases as second/third-line treatment options and use may increase with subsequent approvals by health authorities. The current use of targeted therapies further along in the patient journey for a subset of refractory patients is consistent with the observed distribution in lines of therapy received where 57% of 100 evaluable patients relapsed after first-line treatment and 20% had received 3 or more lines of therapy. It would be interesting to understand if any common “decision” time points exist for when refractory patients move from 1 line of therapy to the next, but further research would be needed to generate a clearer picture of the patient journey in pLGG. A recent analysis of adult survivors of pLGG (*n* = 2501), diagnosed between 1970–1999, assessed temporal changes in treatment concluding that survivors from more recent eras were at lower risk of late mortality (≥5 years from diagnosis), severe chronic health conditions, and subsequent neoplasms.^45^ Although changes in therapy over the decades may account for a portion of the reduced risk observed, the authors proposed that improved screening and survivorship care over these eras may have contributed to improved outcomes by allowing earlier interventions and mitigation of late complications of therapy.^45^ Given the high survival rates in children, adolescents, and young adults with pLGG, treatment strategies in childhood should not only focus on durable disease control but, importantly, on aggressively reducing long-term toxicity and morbidities.^10,46,47^ Treatment guidelines specific to pLGG are lacking and would be beneficial to better guide decision-making, limit the need for emergency treatment where possible, and educate HCPs on new treatment options.^48^

### Limitations

The retrospective claims-based nature of this analysis presents several limitations. First, the heterogeneity of pLGG complicates the use of ICD-10 codes to identify eligible patients and to classify disease and patient characteristics adequately. In particular, it was not possible to: characterize the tumors histologically or genomically, ascertain the patient’s history of disease, anatomical primary site, or determine their stage on the treatment journey. Additionally, the age distribution of patients in this cohort was skewed towards the upper age range (median of 11 years of age [range: 2–18]), which likely introduced bias as infantile and early childhood presentations, such as hypothalamic LGG, are excluded. Second, understanding the sequence of HRU in patients with pLGG was limited by the cross-sectional nature of the study. The ability to obtain longitudinal data on each patient was limited given the likelihood that patients had their initial diagnosis at a tertiary care center and subsequent care at other centers, which may not have been captured in the data set analyzed. This limitation may consequently result in underreporting of HRU in our analysis. Third, the claims data and physician notes on which this analysis was based were created and collected for administrative purposes only, and so there may be issues with misclassification, misinformation, or lack of detailed medical history. However, only patients with the necessary information to conduct the analyses were included in the final sample, which decreased the impact of missing information, but potentially created a selection bias. To mitigate these limitations, we used a comprehensive, robust, and well-utilized national database, which provided access to patient data from many centers and the study design allowed for a relatively long follow-up in identified cases.

Finally, although not designed to be a cost analysis study, the extensive HRU described in the 3-year analysis period suggests that it is likely to be associated with significant cost, particularly as a chronic disease often with additional health conditions developing over time. Our study did not directly measure cost or time spent out of work/school, but our findings suggest an additional burden on patients and families. Mitigating financial hardship for families affected by pLGG is likely to improve treatment adherence, as well as improve socioeconomic and health outcomes and QOL for the entire family.^49,50^

## Conclusions

To our knowledge, this pilot study presents the first US-claims-based analysis of administered medication and HRU specific to patients with pLGG. Here, we show that chronic health issues, arising from both the disease itself and associated treatments, are common burdens for survivors of pLGG. Our results add to the existing body of evidence and begin to characterize how and when this burden manifests and evolves, including the impact on the healthcare system. Despite being considered a “benign” tumor, patients with pLGG have complex healthcare needs that require long-term medical care and high levels of HRU to treat their disease and related sequalae, which can persist for many years. Importantly, high survival rates in pLGG should not obscure the potentially significant and long-term toxicities, functional deficits, and reduced QOL experienced by patients. Interventions to better support families affected by pLGG are important. Larger more intensive studies are needed to provide additional granularity in our understanding of the long-term disease- and treatment-related burden and to inform evidence-based healthcare planning. Development of pLGG treatment guidelines and new treatment options that minimize the burden on patients, as well as on healthcare systems, are needed.

## Supplementary material

Supplementary material is available online at *Neuro-OncologyPractice* (https://academic.oup.com/neuro-oncology).

npae037_suppl_Supplementary_Figure_S1

npae037_suppl_Supplementary_Figure_S2

npae037_suppl_Supplementary_Table_S1

npae037_suppl_Supplementary_Table_S2
