# Supplementary material for: Disease burden and healthcare utilization in pediatric low-grade glioma: A United States retrospective study of linked claims and electronic health records
Source: Neurooncol Pract. 2024 Apr 27;11(5):583–92. doi: 10.1093/nop/npae037 (PMC11398936; doi:10.1093/nop/npae037)
Supplement: npae037_suppl_Supplementary_Figure_S1 [file npae037_suppl_supplementary_figure_s1.pptx]

## Slide 1
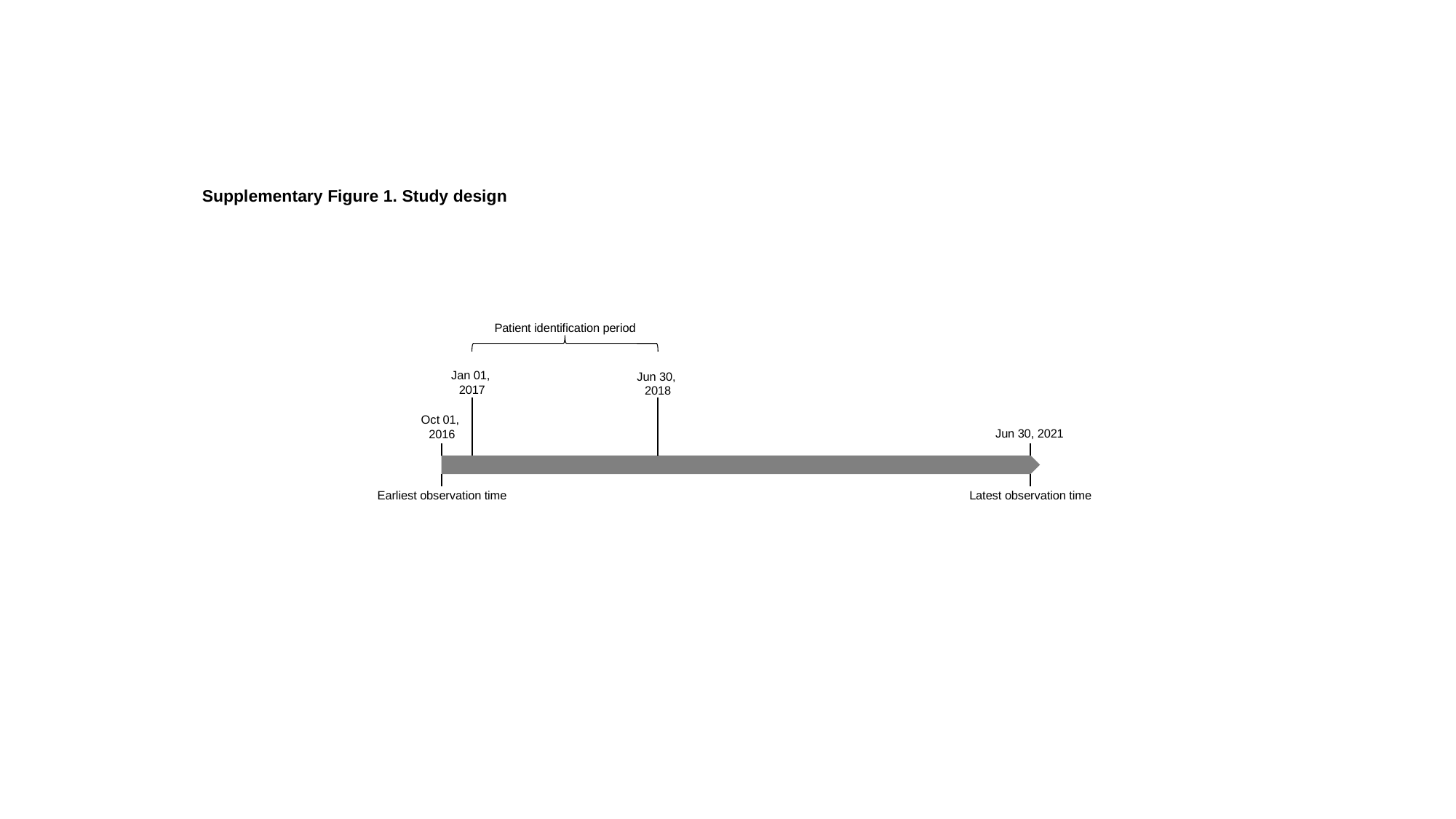

Supplementary Figure 1. Study design
Patient identification period
Jan 01,
2017
Jun 30,
2018
Oct 01,
2016
Jun 30, 2021
Earliest observation time
Latest observation time
