# Supplementary material for: Disease burden and healthcare utilization in pediatric low-grade glioma: A United States retrospective study of linked claims and electronic health records
Source: Neurooncol Pract. 2024 Apr 27;11(5):583–92. doi: 10.1093/nop/npae037 (PMC11398936; doi:10.1093/nop/npae037)
Supplement: npae037_suppl_Supplementary_Figure_S2 [file npae037_suppl_supplementary_figure_s2.pptx]

## Slide 1
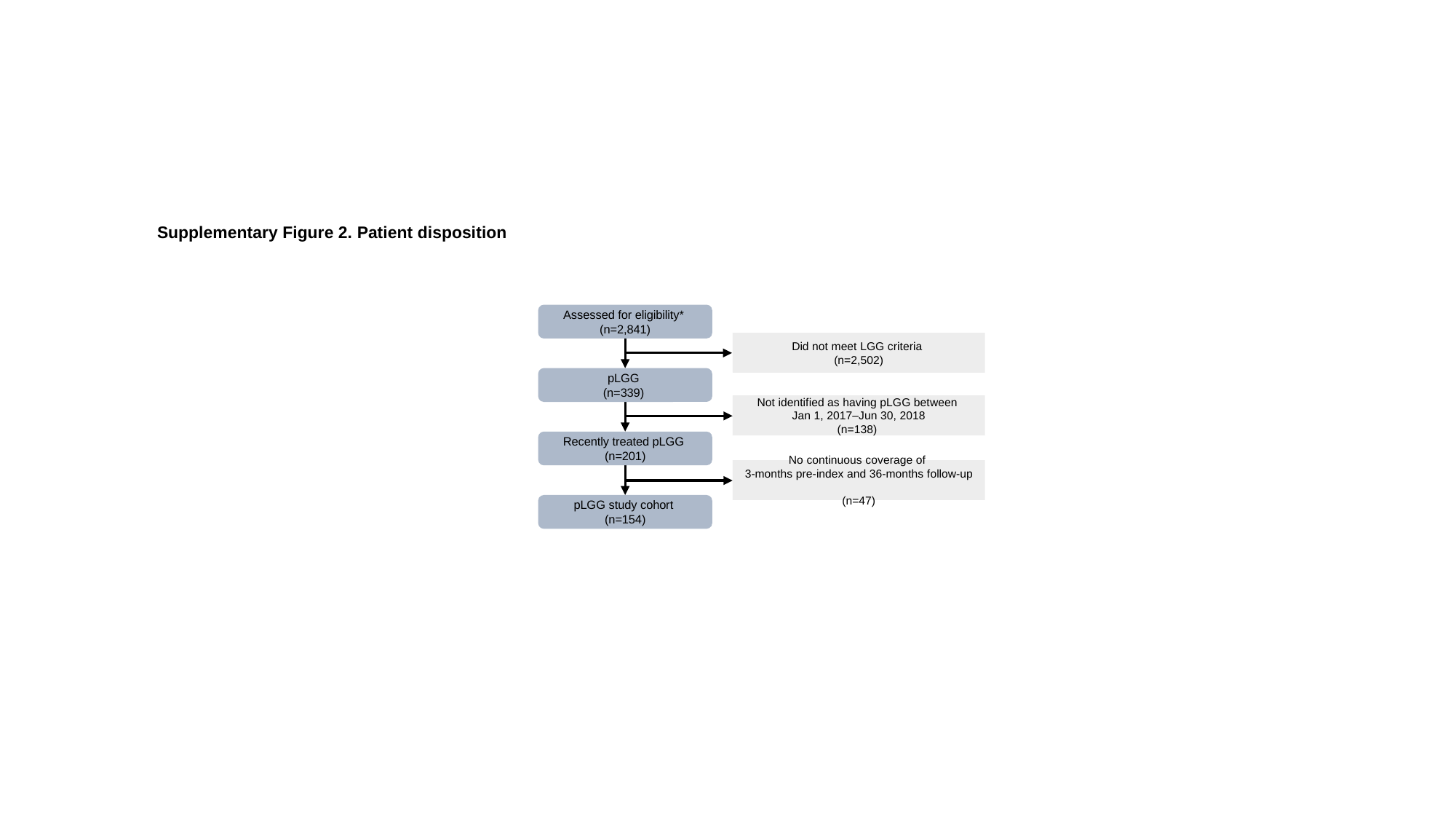

Supplementary Figure 2. Patient disposition
Assessed for eligibility*
(n=2,841)
Did not meet LGG criteria
(n=2,502)
pLGG
(n=339)
Not identified as having pLGG between
Jan 1, 2017–Jun 30, 2018
(n=138)
Recently treated pLGG
(n=201)
No continuous coverage of
3-months pre-index and 36-months follow-up
(n=47)
pLGG study cohort
(n=154)
