# Supplementary material for: Disease burden and healthcare utilization in pediatric low-grade glioma: A United States retrospective study of linked claims and electronic health records
Source: Neurooncol Pract. 2024 Apr 27;11(5):583–92. doi: 10.1093/nop/npae037 (PMC11398936; doi:10.1093/nop/npae037)
Supplement: npae037_suppl_Supplementary_Table_S1 [file npae037_suppl_supplementary_table_s1.docx]

**Supplementary Table 1. ICD-10 codes for brain neoplasms**

| **ICD-10 code** | **Description** |
| --- | --- |
| C71 | Malignant neoplasm of brain |
| C71.0 | Malignant neoplasm of cerebrum, except lobes and ventricles |
| C71.1 | Malignant neoplasm of frontal lobe |
| C71.2 | Malignant neoplasm of temporal lobe |
| C71.3 | Malignant neoplasm of parietal lobe |
| C71.4 | Malignant neoplasm of occipital lobe |
| C71.5 | Malignant neoplasm of cerebral ventricle |
| C71.6 | Malignant neoplasm of cerebellum |
| C71.7 | Malignant neoplasm of brain stem |
| C71.8 | Malignant neoplasm of overlapping sites of brain |
| C71.9 | Malignant neoplasm of brain, unspecified |
| C72.3 | Malignant neoplasm of optic nerve |
| C72.30 | Malignant neoplasm of unspecified optic nerve |
| C72.31 | Malignant neoplasm of right optic nerve |
| C72.32 | Malignant neoplasm of left optic nerve |
| D33.0 | Benign neoplasm of brain, supratentorial |
| D33.1 | Benign neoplasm of brain, infratentorial |
| D33.2 | Benign neoplasm of brain and other parts of CNS |
| D43.0 | Neoplasm of uncertain behavior of brain, supratentorial |
| D43.1 | Neoplasm of uncertain behavior of brain, infratentorial |
| D43.2 | Neoplasm of uncertain behavior of brain, unspecified |
| D49.6 | Neoplasm of unspecified behavior of brain |

ICD-10, International Classification of Diseases 10th Revision.
