# Supplementary material for: Disease burden and healthcare utilization in pediatric low-grade glioma: A United States retrospective study of linked claims and electronic health records
Source: Neurooncol Pract. 2024 Apr 27;11(5):583–92. doi: 10.1093/nop/npae037 (PMC11398936; doi:10.1093/nop/npae037)
Supplement: npae037_suppl_Supplementary_Table_S2 [file npae037_suppl_supplementary_table_s2.docx]

**Supplementary Table 2. Glioma search terms for extraction of pLGG cases using NLP from physician notes**

| **Search terms for glioma in physician notes** | | |
| --- | --- | --- |
| Angiocentric Glioma | MVNT | |
| Angio-centric Glioma | Neuronal glial | |
| Astrocyte | Neuronal-glial | |
| Astrocytoma | Oligo dendrocyte | |
| Chordoid Glioma of 3rd Ventricle | Oligo dendroglia | |
| Chordoid Glioma of III Ventricle | Oligo dendroglioma | |
| Chordoid Glioma of Third Ventricle | Oligodendrocyte | |
| Desmoplastic Infantile Astrocytoma and Ganglioglioma | Oligo-dendrocyte | |
| Desmoplastic Infantile Astrocytoma and Ganglio-glioma | Oligodendroglia | |
| Desmo-plastic Infantile Astrocytoma and Ganglioglioma | Oligo-dendroglia | |
| Desmo-plastic Infantile Astrocytoma and Ganglio-glioma | Oligodendroglioma | |
| Desmoplastic infantile ganglio glioma | Oligo-dendroglioma | |
| Desmo-plastic infantile ganglio glioma | Optic nerve glioma | |
| Desmoplastic infantile ganglioglioma | Optic pathway glioma | |
| Desmoplastic infantile ganglio-glioma | Papillary Glioneuronal | |
| Desmo-plastic infantile ganglioglioma | Papillary Glio-neuronal | |
| Desmo-plastic infantile ganglio-glioma | Papillary Glioneuronal Tumor | |
| Diffuse astrocytoma | Papillary Glio-neuronal Tumor | |
| Diffuse leptomeningeal glioneuronal tumor | Pediatric low-grade glioma | |
| Diffuse leptomeningeal glio-neuronal tumor | Pediatric low-grade glioma | |
| Diffuse lepto-meningeal glioneuronal tumor | Pilocytic astrocytoma | |
| Diffuse lepto-meningeal glio-neuronal tumor | Pilo-cytic astrocytoma | |
| DLGNT | Pilomyxoid astrocytoma | |
| Dysembryoplastic neuroepithelial | Pilo-myxoid astrocytoma | |
| Dysembryoplastic neuro-epithelial | Pleomorphic xantho astrocytoma | |
| Dysembryoplastic neuroepithelial tumor | Pleo-morphic xantho astrocytoma | |
| Dysembryoplastic neuro-epithelial tumor | Pleomorphic xanthoastrocytoma | |
| Ependymal | Pleo-morphic xanthoastrocytoma | |
| Ganglio cytoma | PLGG | |
| Ganglio glioma | PLNTY | |
| Gangliocytoma | Polymorphous Low Grade Neuroepithelial tumor of the Young | |
| Ganglio-cytoma | Polymorphous Low-Grade Neuro-epithelial tumor of the Young | |
| Ganglioglioma | Polymorphous Low Grade Neuroepithelial tumor of Young | |
| Ganglio-glioma | Polymorphous Low-Grade Neuro-epithelial tumor of Young | |
| Glioma | | Polymorphous Low-Grade Neuroepithelial tumor of the Young |
| Glioma of 2nd cranial nerve | | Polymorphous Low-Grade Neuro-epithelial tumor of the Young |
| Glioma of optic nerve | | Polymorphous Low-Grade Neuroepithelial tumor of Young |
| Glioma of optic pathway | | Polymorphous Low-Grade Neuro-epithelial tumor of Young |
| Glioneuronal | | Rosetted glioneuronal |
| Glio-neuronal | | Rosetted glio-neuronal |
| Low grade glioma | | Rosetted glioneuronal tumor |
| Low-grade glioma | | Rosetted glio-neuronal tumor |
| Microglia | | Rosette-forming Glioneuronal |
| Mixed glioneuronal | | Rosette-forming glio-neuronal |
| Mixed glio-neuronal | | Rosette-forming glioneuronal tumor |
| Mixed neuronal glial | | Rosette-forming glio-neuronal tumor |
| Mixed neuronal-glial | | SEGA |
| Multinodular and vacuolating neuronal | | Subependymal astrocytoma |
| Multi-nodular and vacuolating neuronal | | Sub-ependymal astrocytoma |
| Multinodular and vacuolating neuronal tumor | | Subependymal giant cell astrocytoma |
| Multi-nodular and vacuolating neuronal tumor | | Sub-ependymal giant cell astrocytoma |

NLP, natural language processing.
